# Development of a novel humanized anti-TSLP monoclonal antibody HZ-1127 with anti-allergic diseases and cancer potential

**DOI:** 10.1093/abt/tbae006

**Published:** 2024-02-27

**Authors:** Xiaolei Liu, Jianzhong Han, Qian Wang, Peng Wang, Li Li, Kehe Du, Fengchao Jiang, Pei Zhang, Hongjun Liu, Jian Huang

**Affiliations:** Department of Medicine, Perelman School of Medicine at the University of Pennsylvania, 3500 N Broad St, Philadelphia, PA 19140, USA; Department of Research, Coriell Institute for Medical Research, 403 Haddon Ave, Camden, NJ 08103, USA; Department of Research, IPHASE Therapeutic Ltd., 422 Industrial Dr. North Wales, PA 19454, USA; Department of Research, Coriell Institute for Medical Research, 403 Haddon Ave, Camden, NJ 08103, USA; Department of Research, IPHASE Therapeutic Ltd., 422 Industrial Dr. North Wales, PA 19454, USA; Department of Research, IPHASE Therapeutic Ltd., 422 Industrial Dr. North Wales, PA 19454, USA; Department of Research, IPHASE Therapeutic Ltd., 422 Industrial Dr. North Wales, PA 19454, USA; Department of Research, IPHASE Therapeutic Ltd., 422 Industrial Dr. North Wales, PA 19454, USA; Department of Research, IPHASE Therapeutic Ltd., 422 Industrial Dr. North Wales, PA 19454, USA; Department of Research, Coriell Institute for Medical Research, 403 Haddon Ave, Camden, NJ 08103, USA; Department of Center for Metabolic Disease Research, Temple University Lewis Katz School of Medicine, 3500 N Broad St, Philadelphia, PA 19140, USA; Department of Biomedical Sciences, Cooper Medical School of Rowan University, 303 Cooper St, Camden, NJ 08102, USA

**Keywords:** development, novel, Humanized, anti-TSLP, monoclonal antibody, HZ-1127

## Abstract

Thymic stromal lymphopoietin (TSLP) is a member of the IL-2 cytokine family and has been widely recognized as a master regulator of type 2 inflammatory responses at barrier surfaces. Recent studies found dysregulation of the TSLP–TSLP receptor (TSLPR) pathway is associated with the pathogenesis of not only allergic diseases but also a wide variety of cancers including both solid tumors and hematological tumors. Thus, the blockade of TSLP represents an attractive therapeutic strategy for allergic diseases and cancer. In this study, we report the development of a novel humanized anti-TSLP monoclonal antibody (mAb) HZ-1127. Binding affinity, specificity, and ability of HZ-1127 in inhibiting TSLP were tested. HZ-1127 selectively binds to the TSLP cytokine with high affinity and specificity. Furthermore, HZ-1127 dramatically inhibits TSLP-dependent STAT5 activation and is more potent than Tezepelumab, which is an FDA-approved humanized mAb against TSLP for severe asthma treatment in inhibiting TSLP-induced CCL17 and CCL22 chemokines secretion in human peripheral blood mononuclear cells. Our pre-clinical study demonstrates that HZ-1127 may serve as a potential therapeutic agent for allergic diseases and cancer.

## Teaser

Statement of Significance: Our study is the first to introduce the development of a humanized monoclonal antibody called HZ-1127, which exhibits a capacity for selectively binding to the thymic stromal lymphopoietin cytokine with exceptional affinity and specificity.

## INTRODUCTION

Thymic stromal lymphopoietin (TSLP)—which is a member of the four-helix-bundle cytokine family and paralog of the cytokine IL-7—plays a fundamental role in maintaining immune homeostasis and has been linked to the pathogenesis of type 2 inflammatory diseases, including atopic dermatitis (AD), food-hypersensitivity reactions, and asthma [1]. Biological functions of TSLP require heterodimer formation between the TSLP receptor (TSLPR) and IL-7 receptor α-chain, which leads to activation of an intracellular signaling network that includes Janus kinases 1 and 2 (JAK1/2), as well as signal transducers and activators of transcription 5 (STAT5) axis on a broad range of cell types [2, 3].

TSLP is involved in both physiological and pathological immunity-related activities. Activation of the TSLP pathway primes dendritic cells (DC) to induce type 2 inflammation and activates functional type 2 helper T (Th2) cells, innate lymphoid cells (ILCs), macrophages, and other immune cells [1]. Particularly, TSLP stimulates DCs to secrete chemokines like CCL17 and CCL22 [4], which thereby promote Th2 cells transfer from thoracic lymph nodes to the airways [5]. TSLP is thus considered a master regulator of type 2 immune responses at barrier surfaces. In asthmatic patients, expression of TSLP and Th2 cytokines are significantly increased in the airway epithelium and lamina propria [6]. A study using a murine model showed that induction of systemic release of TSLP could drive the development of an asthmatic phenotype after antigen challenge in the lungs, and blockade of TSLP signaling rescued the development of asthma [7]. The therapeutic potential of inhibiting TSLP in patients with asthma was also assessed by using Tezepelumab, which is a fully humanized monoclonal antibody (mAb) against TSLP that has been approved by the FDA for severe asthma treatment in the U.S. under the brand name of Tezspire in 2021. In the 52-week trial of moderate-to-severe uncontrolled asthma, treatment with Tezepelumab significantly reduced the annualized asthma exacerbation rate in both patients with type 2 asthma as well as those with non-type 2 asthma [8]. Interestingly, recent advances in TSLP biology have revealed the unexpected role of TSLP in the induction and development of a wide variety of cancers (reviewed in [9]), suggesting the potential for therapeutic intervention of both allergic diseases and cancer through modulation of the TSLP pathway and a clear need for additional therapeutic approaches to target TSLP.

In this study, we explore the distinctions in the mechanisms of action (MOA) between anti-TSLP and anti-TSLPR antibodies. While anti-TSLP antibodies target the cytokine thymic stromal lymphopoietin (TSLP) itself, thereby preventing its interaction with its receptor complex, anti-TSLPR antibodies specifically target the TSLP receptor (TSLPR), impeding the TSLP signaling cascade at a different point. This distinction is crucial, as TSLP plays a pivotal role in initiating allergic inflammation and immune responses, and the precise inhibition point can significantly influence therapeutic outcomes. Understanding these differences is vital for developing targeted therapies for conditions such as asthma, allergic rhinitis, and atopic dermatitis, where TSLP-mediated pathways are key contributors to the pathogenesis.

Previously, we reported a development and characterization of a novel humanized anti-interleukin-6 antibody HZ0408b with anti-rheumatoid arthritis therapeutic potential [10]. In this study, we employed a similar approach to develop a novel humanized anti-TSLP mAb HZ-1127, which selectively binds to the TSLP cytokine with high affinity and specificity. Furthermore, HZ-1127 dramatically inhibits TSLP-dependent STAT5 activation and is more potent than Tezepelumab in inhibiting TSLP induced CCL17 and CCL22 chemokines secretion in human peripheral blood mononuclear cells (PBMCs). Thus, HZ-1127 may serve as a potential therapeutic strategy for allergic diseases and cancer.

## MATERIALS AND METHODS

### Generation and humanization of HZ-1127

In this study, the mouse-immunization was used to generate the antibodies. All animal experiments were carried out according to the procedures approved by the Institutional Animal Care and Use Committee of IPHASE Therapeutic Ltd and were in compliance with all relevant ethical regulations and guidelines set forth by the National Institutes of Health. HZ-1127 (iPhase, Cat# 062A031.02) is a genetically engineered monoclonal antibody, humanized from a mouse anti-human TSLP antibody using complementarity-determining region (CDR) grafting method (The sequences of HZ-1127 are shown in the Supplementary Fig. S1). Hybridomas were generated using standard protocols. In brief, Balb/c mice were immunized with purified recombinant rhTSLP-His or rhTSLP-mFc (IgG) fusion protein. Titers were assessed, and the spleen cells were fused with SP2/0 cells. Hybridomas were selected and supernatants from the resulting clones were screened by enzyme-linked immunosorbent assay (ELISA) for TSLP binding affinity and neutralizing activity. One of the positive clones was obtained and designated as HZ-1127. HZ-1127 variable regions were then cloned onto a human full-length IgG (IgG1 and IgG2) framework and subsequently humanized and engineered to minimize interactions with the immune system. Humanization was performed by grafting the CDRs into the closest human variable (V) region light- and heavy-chain framework sequences as previously described [11].

### Cell culture

HEK293T (ATCC #CRL-3216) cells were cultured in Dulbecco's Modified Eagle Medium (DMEM) (ATCC #30–2002) supplemented with 10% fetal bovine serum (Hyclone #SH30071.03) and 1% penicillin/streptomycin (GIBCO #15140). Human PBMCs (Human Immunology Core at the University of Pennsylvania) were cultured in RPMI1640 (Gibco, Cat# 11875093) supplemented with 10% fetal bovine serum, 1% penicillin/streptomycin, and 1% 200 mM l-Glutamine (ATCC, Cat# 30–2214). Cells were maintained at 37 °C and 5% CO_2_.

### Cell surface antigen-binding assay

Binding activity to TSLP and inhibitory activity of TSLP binding to TSLPR were measured by ELISA as previously described [13]. Briefly, 96-well plates were coated with 1 μg/ml TSLP-hFc fusion protein (iPhase, Cat# 051A12.22) in phosphate-buffered saline (PBS) overnight at 4 °C. After blocking for 1 hr with 0.4% BSA in PBS at room temperature, increasing concentrations of humanized anti-TSLP mAbs HZ-1127 and Tezepelumab (iPhase, Cat# 062A051.02) were added into the plates at room temperature for 2 hr. For TSLP and TSLPR interaction blocking assay, 96-well plates were coated with 1.5 μg/ml of TSLP-hFc fusion protein in PBS for 16 hr at 4 °C. A total of 1 μg/ml of TSLPR-mFc protein was added either in the absence or presence of increasing concentrations of humanized anti-TSLP mAbs HZ-1127 and Tezepelumab at room temperature for 2 hr. Plates were subsequently washed three times and incubated with an HRP-conjugated anti-His secondary antibody for 1 hr at room temperature. After washing, plates were developed with TMB. The OD was measured at 450 nM. Each sample was tested in triplicate. Competition ELISA of HZ-1127 and Tezepelumab binding to TSLP-hFc were analyzed by competitions with an equal amount of limiting diluted concentrations of HZ-1127 and Tezepelumab at 12.5 and 3.125 μg/ml, respectively. Cytokines used for cross-reactivity assay were purchased from Sinobiological (The catalog numbers are shown in the Supplementary Table S1). ELISA was performed to measure secretion of CCL17 (R&D, DY364) and CCL22 (R&D, DMD00) from human PBMCs according to the manufacture’s instruction.

### Binding affinity

The binding affinity of humanized anti-TSLP mAbs against TSLP was measured using Bio-layer Interferometry (BLI) by FroteBio Blitz (Pall, USA). Anti-human Fc Capture biosensors (Cat No, 18–5060, Fortebio, Pall) were used to probe purified antibodies at concentrations from 100 to 400 nM. The association and disassociation kinetics of binding to TSLP-hFc were measured using the following setting: initial baseline for 60 secs, followed by loading of antibodies for 120 secs, baseline for 60 secs, association for antigen for 120 secs, and dissociation for 120 secs. The binding data were globally fitted to a 1:1 binding model to calculate the equilibrium dissociation constant (KD), association constant (Ka), and dissociation constant (Kd).

### Immunoblot assay

To determine the type of epitope for HZ-1127, 30 μg heated TSLP-A/S-His protein was subjected to odium dodecylsulfate polyacrylamide gel electrophoresis (SDS-PAGE) and HZ-1127 or Tezepelumab was used as primary antibody for immunoblot assay as described previously.

### Statistical analysis

Calculation of relative potencies of the antibodies was performed in GraphPad Prism 8 software with a sigmoidal dose–response model. All data are presented as mean ± SD.

## RESULTS

### Characterization of the mAb HZ-1127

**Figure 1:**
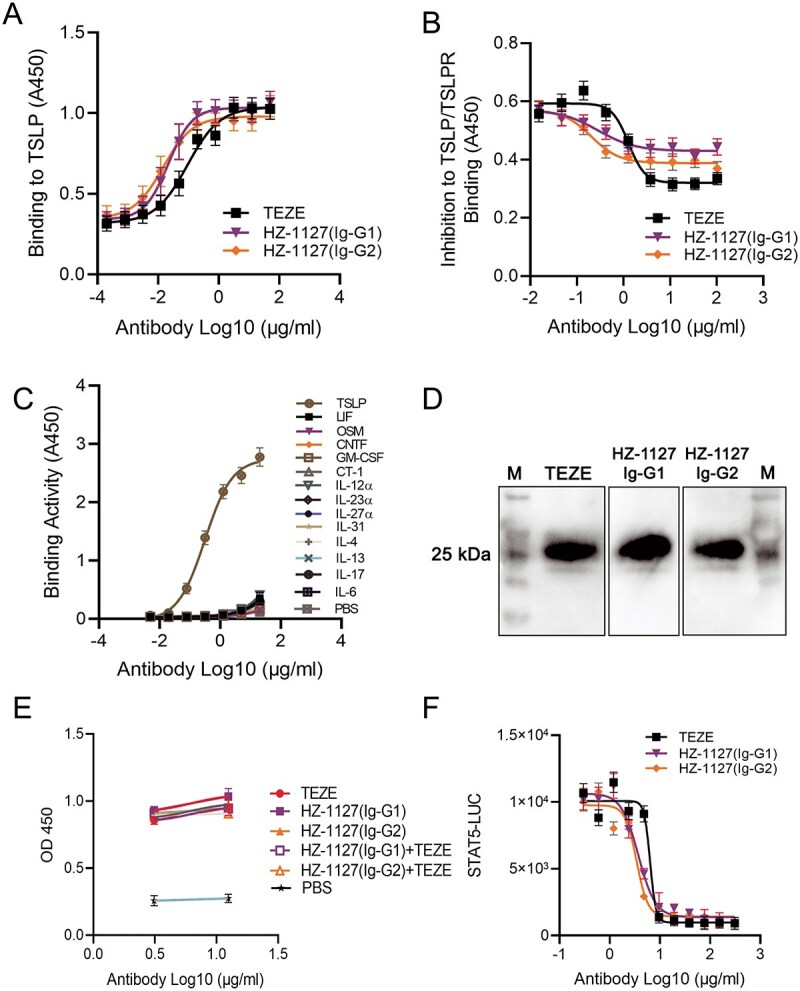
Characterization of the mAb HZ-1127. (A) The binding activity of the humanized anti-TSLP mAbs HZ-1127 (Ig-G1/Ig-G2) and Tezepelumab to TSLP was measured by ELISA using an HRP-conjugated anti-human IgG secondary antibody. Each sample was assayed in triplicates. Mean absorbance at 450 nm is presented. (B) The inhibitory effect of HZ-1127 (Ig-G1), HZ-1127 (Ig-G2), and Tezepelumab on TSLP and TSLPR binding was measured by ELISA. Each sample was performed in triplicates. (C) Cross-reactivity of HZ-1127 to other cytokines was measured by ELISA as previously described in Fig 1A. (D) Immunoblot assay to determine the epitope type of HZ-1127 and Tezepelumab. 30 μg heated TSLP-A/S-His protein was subjected to SDS-PAGE and HZ-1127 or Tezepelumab was used as primary antibody for immunoblot assay. M, protein marker. TEZE, Tezepelumab. (E) Competition ELISA of HZ-1127 (Ig-G1/Ig-G2) and Tezepelumab binding to TSLP-A/S-His were analyzed by competitions with an equal amount of limiting diluted concentrations of HZ-1127 and Tezepelumab at 12.5 and 3.125 μg/ml, respectively. (F) Inhibition of TSLP dependent STAT5 activation. HEK293F cells that were transfected with constructs expressing hTSLPR, hIL7Rα, and pGL4.52 [luc2P/STAT5 RE/Hygro] were treated with the combination of TSLP-hFc recombinant protein and the limiting diluted anti-TSLP antibodies, each group was tested in triplicate.

First, we conducted ELISA to assess the antigen-binding activity of the humanized anti-TSLP mAb HZ-1127. We used Tezepelumab, which is an FDA-approved anti-TSLP mAb under the brand name of Tezspire for the treatment of asthma as a control (Fig. 1A). Both HZ-1127 and Tezepelumab are effective in binding to TSLP-hFc in a dose-dependent manner. EC_50_s of HZ-1127-IgG1 and HZ-1127-IgG2 are 0.023 and 0.015 μg/ml respectively, while EC_50_s of Tezepelumab are 0.092 μg/ml (Fig. 1A). We next measured the antigen-binding affinity of HZ-1127 using Bio-layer Interferometry (ForteBio BLItz). HZ-1127 bound to human TSLP antigen with a KD of 3.66 ×10^−8^ M, which is similar to Tezepelumab with a KD of 3.86×10^−8^ M.

We then tested the ability of the humanized mAbs in blocking the interaction between TSLP and TSLPR. We coated 96-well plates with recombinant TSLP-hFc and measured the binding of TSLPR-mFc protein. As shown in Fig. 1B, TSLP bound TSLPR without the presence of the antibodies; however, the binding activity was blocked in a dose-dependent manner when the humanized anti-TSLP mAbs were added. While the maximum inhibition achieved by HZ-1127-IgG1 and HZ-1127-IgG2 is marginally lower compared to Tezepelumab, their IC50 values of 0.31 and 0.21 μg/ml, respectively, are significantly lower than Tezepelumab’s IC50 of 1.28 μg/ml (Fig. 1B), indicating a higher potency of HZ-1127 variants in terms of half-maximal inhibitory concentration.

We further measured the cross-reactivity of HZ-1127 to other cytokines. ELISA results demonstrate that HZ-1127 does not have binding activity to cytokines LIF, OSM, CNTF, GM-CSF, CT-1, IL-12α, IL-23α, IL-27α, IL-31, IL-4, IL-13, IL-17, and IL-6 (Fig. 1C), suggesting that HZ-1127 is highly specific in inhibiting TSLP activity. Overall, these results suggest that HZ-1127 has potent binding and neutralizing activity to human TSLP with high specificity and low cross-reactivity, thereby impeding the human TSLP-TSLPR interaction.

### Epitope analysis of the antibody HZ-1127

We then performed an immunoblot assay to determine the epitope type of HZ-1127. Briefly, we used the heat-denatured TSLP-A/S-His protein for SDS-PAGE immunoblot assay and used HZ-1127 and Tezepelumab as primary antibodies. Our results showed that both HZ-1127 and Tezepelumab recognized the linear epitope of TSLP (Fig. 1D). The competition ELISA involving HZ-1127 and Tezepelumab was subsequently performed. In this assay, TSLP-mFc or TSLP-A/S-His was used to coat a 96-well plate, followed by the addition of HZ-1127 in a limiting dilution, either alone or in conjunction with an equivalent quantity of Tezepelumab. The outcomes of this experiment indicated that both antibodies, HZ-1127 and Tezepelumab, bind to an epitope on TSLP that is either identical or highly similar (Fig. 1E).

### Inhibition of TSLP dependent STAT5 activation

Next, we tested the inhibition of TSLP-dependent STAT5 activation using a STAT5-Luc reporter. HEK293F cells that were transfected with constructs expressing hTSLPR, hIL7Rα, and pGL4.52 [luc2P/STAT5 RE/Hygro] were treated with the combination of TSLP-hFc recombinant protein and the limiting diluted anti-TSLP antibodies. As shown in Fig. 1F, IC_50_s of HZ-1127-IgG1 and HZ-1127-IgG2 effective in inhibiting STAT5 activity are 3.6 and 3.33 μg/ml and that of Tezepelumab is 6.21 μg/ml, indicating that HZ-1127 is more potent in inhibiting TSLP-induced STAT5 signaling activation than Tezepelumab (Fig. 1F).

### Inhibition of TSLP induced CCL17 and CCL22 secretion in PBMCs

**Figure 2:**
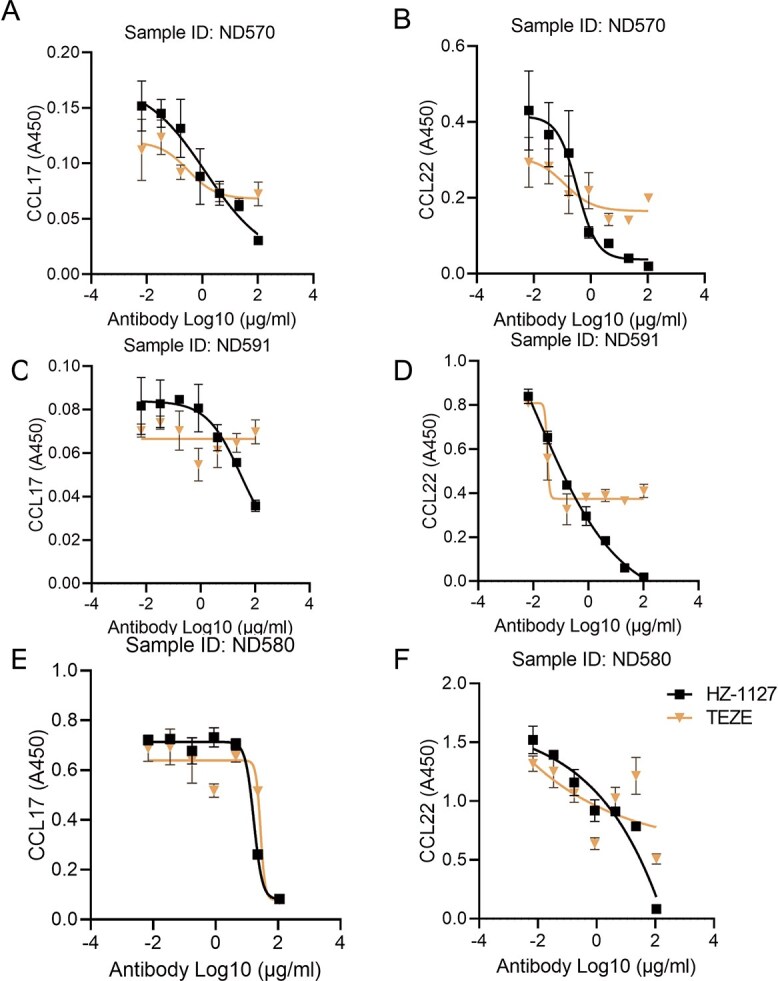
Inhibition of TSLP induced CCL17 and CCL22 secretion in PBMCs. Human PBMCs (5 × 10^5^) were plated in a 96-well plate and incubated with serially diluted humanized anti-TSLP mAbs ZH-1127 or Tezepelumab in the presence of 50 ng/ml recombinant human TSLP-hFc for 24 hr. Supernatants were then collected for ELISA according to the manufacturer’s instruction, each sample was tested in triplicate.

We carried out further investigations to assess if the humanized anti-TSLP monoclonal antibody (mAb) could suppress the secretion of CCL17 and CCL22 in PBMCs triggered by TSLP. It was observed that TSLP-hFC at a concentration of 50 ng/ml successfully induced the secretion of CCL17 and CCL22. Moreover, both our humanized anti-TSLP mAb, HZ-1127, and Tezepelumab effectively inhibited this induction in a dose-dependent manner, as evidenced by experiments conducted with PBMCs from three different donors (Fig. 2). Notably, there were observable sample-to-sample variations, particularly evident in ND591 samples (Fig. 2 panel C), which highlight the variability in response across different samples. In conclusion, compared with Tezepelumab, our humanized anti-TSLP mAb HZ-1127 is significantly potent in inhibiting CCL17 and CCL22 secretion.

## DISCUSSION

**Figure 3:**
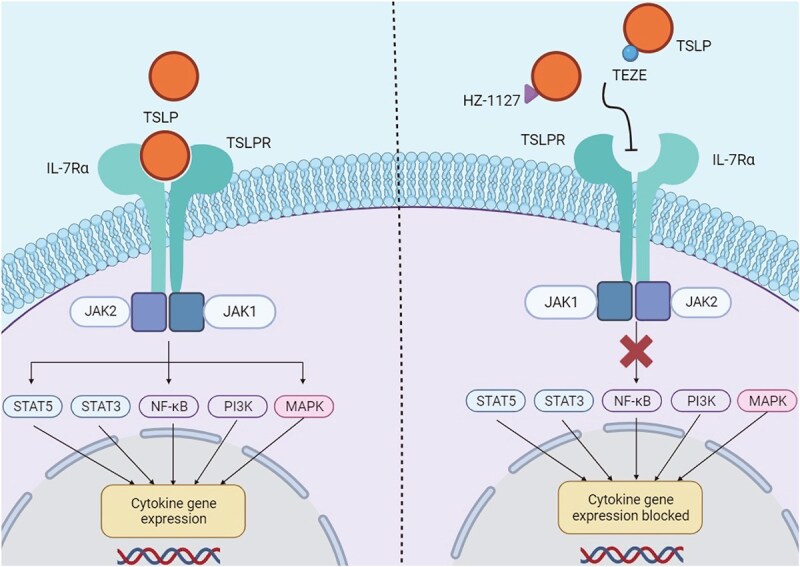
The diagram of the inhibition of TSLP Signaling by HZ-1127 and TEZE. The left panel depicts the canonical TSLP signaling pathway initiated by TSLP binding to its receptor complex, consisting of TSLPR and IL-7Rα on the cell surface. This interaction activates the JAK1/JAK2 pathway, leading to the phosphorylation and nuclear translocation of STAT5 and STAT3, which ultimately results in cytokine gene expression and subsequent inflammatory response. Right panel demonstrates the inhibitory action of HZ-1127, and TEZE as a positive control, on TSLP signaling. HZ-1127 effectively blocks the binding of TSLP to its receptor, thereby preventing the downstream activation of the JAK/STAT pathway, NF-κB, PI3K, and MAPK pathways, culminating in the suppression of cytokine gene expression and the mitigation of the inflammatory response.

In this study, we have focused on the development and characterization of HZ-1127, an anti-TSLP antibody. It is pertinent to discuss the differences in the MOA between antibodies targeting TSLP, like HZ-1127, and those targeting the TSLP receptor (TSLPR). Anti-TSLP antibodies, including HZ-1127, function by directly neutralizing TSLP, thereby preventing it from interacting with its receptor complex, TSLPR-IL-7a. This neutralization effectively inhibits the downstream signaling pathways activated by TSLP, reducing its role in immune modulation and inflammation. In contrast, anti-TSLPR antibodies target the receptor complex itself. By blocking the interaction site for TSLP on TSLPR, these antibodies can selectively disrupt the signaling cascade initiated by TSLP binding, offering a more targeted approach to modulating the TSLP pathway. This difference in MOA is crucial, as it dictates the antibodies’ therapeutic applications and potential side-effect profiles. While both approaches aim to modulate TSLP-mediated pathways, the direct targeting of TSLP by antibodies like HZ-1127 could offer broader therapeutic potential in diseases where TSLP plays a pivotal role, such as asthma, allergic rhinitis, and atopic dermatitis (Fig. 3).

In recent years, remarkable advances have been made in translating the biology of TSLP to the treatment of patients with various allergic diseases including asthma (See Supplementary Table S2 For the list of typical anti-TSLP antibodies under clinical development). For patients with mild or moderate asthma, the disease could be well controlled by inhaled corticosteroids with a combination of long-acting β2-adrenergic agonists, or additional medications such as leukotriene modifiers and oral corticosteroids. However, severe asthma can remain uncontrolled and requires add-on treatments such as mAbs directly targeting Th2 cytokines IL-4, IL-5 as well as their receptors [12–14]. TSLP locates at the very upstream level of the complex network of cellular and molecular pathways leading to airway inflammation. Thus, TSLP blockade with Tezepelumab represents an important advancement for severe asthma treatment. Indeed, in clinical trials, the most exciting finding is that Tezepelumab could prevent exacerbations in both type 2 asthma as well as non-type 2 asthma [8, 15, 16]. In this regard, the clinical efficacy of TSLP blockade in severe asthma patients could not be solely explained by the effects of the anti-TSLP mAbs on DCs and Th2 cells. Further study is required to assess the biological functions of anti-TSLP mAbs in additional cell types such as basophils and the non-immune/inflammatory cells such as neurons and the airway structural cells.

In our competition ELISA experiments, we observed similar optical density (OD450) readings across all five groups when HZ-1127 was tested both alone and in combination with an equal amount of Tezepelumab. This similarity in OD450 readings, despite the presence of competing antibodies, suggests that HZ-1127 and Tezepelumab recognize a similar or the same epitope on TSLP. When two antibodies bind to the same or overlapping epitopes, the addition of one does not significantly alter the binding affinity of the other, as evidenced by the consistent OD450 readings across different conditions. This phenomenon indicates competitive inhibition, where the binding of one antibody to the epitope hinders or does not significantly enhance the binding of the other, implying their interaction with the same epitopic region (Fig. 3). To further substantiate this conclusion, additional experiments, such as epitope mapping, need to be carried out for more precise determination of the epitope specificity.

TSLP also has been found to exert an essential role in the induction and progression of a variety of tumors. One study demonstrated that the TSLP pathway contributes to the progression of metastatic breast cancer by promoting a Th2-type tumor microenvironment to blunt anti-tumor immunity [9, 17]. However, its role as a pro- or anti-tumor factor is still controversial and seems tumor type and context dependent. Expanding research into the role of TSLP in different tumor types is urgently required to inform the application of anti-TSLP therapies in cancer.

In this study, we developed a novel humanized anti-TSLP mAb HZ-1127. We demonstrate that (1) HZ-1127 selectively binds to human TSLP and inhibits TSLP binding to TSLPR in a dose-dependent manner, (2) HZ-1127 inhibits the induction of STAT5 activation by TSLP, and (3) HZ-1127 is more potent than Tezepelumab in inhibiting TSLP induced TARC/CCL17 and MDC/CCL22 chemokines secretion in PBMCs. Our pre-clinical study paved the way for future in vivo studies to test the efficacy and the range of anti-TSLP antibody HZ-1127 for allergic diseases and cancer treatment.

## Supplementary Material

Sup_Fig1_tbae006

Supplementary_Table_1_tbae006

Supplementary_Table_2_tbae006

## Data Availability

The data supporting this article will be made available upon reasonable request to the corresponding authors.

## ABBREVIATIONS

TSLP, Thymic stromal lymphopoietin; JAK1/2, Janus kinases 1 and 2; STAT5, Signal transducer and activator of transcription 5; mAb, Monoclonal antibody; PBMC, peripheral blood mononuclear cells; DC, dendritic cells; Th2, type 2 helper T cells; ILCs, Innate lymphoid cells.

## ANIMAL RESEARCH STATEMENT

Mouse immunization procedures were reviewed and approved by the Institutional Animal Care and Use Committee (IACUC) of IPHASE Therapeutic Ltd and were in compliance with all relevant ethical regulations and guidelines set forth by the National Institutes of Health (NIH). Details are covered in the Methods section.

## CREDIT AUTHOR STATEMENT

Xiaolei Liu (Data curation [Equal], Writing—original draft [Equal], Writing—review & editing [Equal]), Jianzhong Han (Data curation [Equal], Writing—review & editing [Equal]), Qian Wang (Data curation [Equal]), Peng Wang (Writing—review & editing [Equal]), Li Li (Data curation [Equal]), Kehe Du (Data curation [Equal]), Fengchao Jiang (Data curation [Equal]), Pei Zhang (Data curation [Equal]), Hongjun Liu (Conceptualization [Equal], Funding acquisition [Equal], Project administration [Equal], Supervision [Equal]), and Jian Huang (Funding acquisition [Equal], Project administration [Equal], Supervision [Equal], Writing—original draft [Equal], Writing—review & editing [Equal]).
